# Hyaluronic acid/poly(ethylenimine) polyelectrolyte multilayer coatings for siRNA-mediated local gene silencing

**DOI:** 10.1371/journal.pone.0212584

**Published:** 2019-03-19

**Authors:** Olivia Koenig, Bernd Neumann, Christian Schlensak, Hans Peter Wendel, Andrea Nolte

**Affiliations:** Department of Thoracic, Cardiac, and Vascular Surgery, University of Tuebingen, Tuebingen, Baden-Wuerttemberg, Germany; University of Puerto Rico, Rio Piedras Campus, PUERTO RICO

## Abstract

Local gene delivery systems utilizing RNA interference technology are a promising approach for therapeutic applications where site-specific release of agents is desired. Polyelectrolyte multilayers (PEMs) can be constructed using the layer-by-layer (LbL) technique and serve as a depot for bioactive substances, which can then be released in a controlled manner. Multilayers of hyaluronic acid/poly(ethylenimine) HA/PEI were built up with different numbers of bilayers and PEI-siRNA particles were embedded in bioactive layers for gene silencing. The increase of the bilayers and the release of siRNA particles were demonstrated by fluorescence intensity measurement with a fluorescence reader. Two different LbL techniques were tested for the reduction of ICAM–1 expression in EA.hy926: PEM build-up by dipping or drying steps, respectively. Herein, the drying technique of the bioactive layers with ICAM siRNA mediated a significant reduction of the ICAM–1 expression from 3 to 24 bilayers. The fluorescent siRNA release study and the re-culturing of the HA/PEI films demonstrated a release of the transfection particles within the first hour. The advantage of dried built-up PEMs compared to a dried monolayer of PEI-siRNA particles with the same siRNA concentration was a significant higher amount of viable cells.

## 1 Introduction

The RNA interference (RNAi) mechanism is a powerful and specific technique in molecular biology and shows high potential for therapeutic applications, including cardiovascular diseases (CVD), infectious diseases and cancer [1–8]. RNAi was first discovered by Fire *et*. *al* in 1998 in *C*. *elegans* with double-stranded RNA causing the silence of complementary messenger RNA sequence [9]. This self-defense mechanism in *Eukarya* is known for preventing infections and following gene integrity by pathogens and regulating gene expression processes. The ability to artificially produce short interfering RNA (siRNA) with 21–23 nucleotides which binds specific to the target mRNA is a promising approach for various therapeutic applications with the aim of transient gene silencing [10]. Despite promising therapeutic options, the delivery of siRNA to the target tissue or cells is one of the major challenges. Systemic delivery of siRNA is preferred when the target of interest is quite difficult to access and especially in cancer therapy approaches [11–14]. However, if targeted silencing is desired, such as e.g. for vascular stents or general implants, local application of siRNA is essential to avoid the occurrence of systemic off targets and to reduce the amount of active substances, while locally a high release can be guaranteed [1, 3, 15–17].

Vascular cell adhesion molecule 1 (VCAM–1), E-selectin, and Intercellular Adhesion Molecule 1 (ICAM–1) are cell adhesion molecules on endothelial cells which are increasingly expressed in inflammation. Inflammatory processes can be observed in atherosclerotic lesions or after vascular intervention like coronary artery stenting, which in the last case is called in-stent restenosis. This adverse side effect of a neointimal hyperplasia should be avoided in atherosclerotic therapy. A promising approach is the reduction of the inflammatory process by siRNA gene silencing of the above mentioned involved adhesion proteins [18–20]. Reducing inflammation is thought to decrease the probability of restenosis, avoid re-surgeries, and reduce the duration of antithrombotic drug use.

The complexation of the siRNA with transfection agents for better stability benefits the approach of substrate-mediated transfection. These complexes can be incorporated in large quantities in coatings and act as a depot. Herein, the use of polyelectrolyte multilayers (PEMs) is a promising approach in the field of substrate-mediated active substance delivery [1, 21–26]. The ability to incorporate drugs into the PEMs makes this system interesting for siRNA release and the desired gene silencing [1, 27, 28]. A simple technique for the deposition of PEMs is the versatile layer-by-layer (LbL) technique, in which polyelectrolytes are alternately deposited to a substrate. The alternation of positive and negative charged materials creates electrostatic interactions that stabilize the structure. Popular materials for cell biomaterial interaction were used in many PEM research works like the cationic polyelectrolytes: poly(L-lysine), chitosan, and poly(ethylenimine), and the anionic ones: alginate, hyaluronic acid, and polyacrylic acid [29–34]. Among the cationic polymers, the poly(ethylenimine) (PEI) is much favored for the complexation of negatively charged siRNA, as it forms a non-covalent bond with its strong cationic charge density. By complexing siRNA, the limiting factors of nucleic acid transfection, such as negative charge, degradation by nucleases, stimulation of the immune system, can be avoided. PEI has nitrogen on every third atom and is hence protonable in an endosome [35]. Due to its buffer capacity, chloride anions flow into the endosome which causes an influx of water and consequently an osmotic swelling. This so called “proton-sponge-effect” causes the endosome to burst and releases the PEI-siRNA complexes into the cytoplasm [36]. For successful transfection, the choice of molecular weight, chemical structure and nitrogen phosphate (N/P) ratio is important. The N/P ratio is defined as the quotient of the moles of the amine groups of cationic polymer to the phosphate groups of DNA or RNA and is crucial for efficient gene delivery. The ratio determines the size of the particles for transfection and is therefore an important measure for cell experiments [37–39].

Hyaluronic acid (HA) is a negatively charged glycosaminoglycan which occurs naturally like in the extracellular matrix, synovial fluid, umbilical cord, and in blood [40, 41]. The biopolymer properties like biocompatibility, biodegradability, and no immunogenicity make hyaluronic acid a suitable material in wound healing, treatment of knee osteoarthritis or for tissue-engineered scaffolds [42–44]. Crucial for this is the molecular weight of the hyaluronic acid which is responsible for the properties of the polymer. The high molecular weight hyaluronic acid has anti-inflammatory effects during tissue injury, while the low molecular weight hyaluronic acid is responsible for the activation of pro-inflammatory chemokines and cytokines [45]. Although HA is not a typical transfection agent due to its negative charge, it is often used in nucleic acid delivery in combination with chitosan or poly-L-arginine [46, 47]. The combined use of HA with the transfection agent PEI has the great advantage that HA can reduce the cytotoxic effects of PEI [48].

The well considered choice of biomaterials is of particular importance for the success of transfection and the necessary cell adhesion. In addition to parameters such as thickness, roughness, and viscoelasticity of the substrate, stiffness is of great importance for cell adhesion [49, 50]. The cultivation of primary cells on soft biomaterials from natural origin causes low adhesion on the substrate and thus leads to apoptosis in *in vitro* assays [51]. As a result, soft PEM layers cannot be properly assessed for toxicity. It is indistinguishable whether the apoptosis occurs due to the cytotoxicity of the polymer or because of low stiffness. The ‘reverse assay’ is a suitable method to avoid possible influence by the stiffness where coated substrates can be placed on pre-cultured cells [51].

In this study we describe the potential of a PEM system that might be used as a coating for medical devices which is capable to release siRNA from HA/PEI multilayers for ICAM–1 gene knockdown in EA.hy926 in vitro. Bilayers from 3 to 24 were tested in an ‘reverse assay’ for gene knockdown and transfection efficiency using two different LbL techniques, wet or dried layers, on ICAM–1 silencing. The build-up of the (HA/PEI)_n_ multilayers and the release of siRNA particles were determined by fluorescence intensity as a scan of the coated substrates and of the supernatant, respectively. A possible influence of the polymer on cell growth was investigated with the determination of cell viability.

## 2 Materials and methods

### 2.1 Polyelectrolyte solutions for PEM build-up

Branched polyethylenimines (PEI), (molecular weight (MW) = 750 kDa and 25 kDa) were purchased from Sigma-Aldrich (Steinheim, Germany) and prepared in ddH_2_O (Ampuwa, Fresenius Kabi, Bad Homburg, Germany). The concentration of 750 kDa PEI was 1 μM and of 25 kDa 100 μM. Sodium hyaluronate 95% (MW = 1500–2200 kDa) was obtained from Acros Organics (Fisher Scientific, Schwerte, Germany) and 1 mg/mL was dissolved in 5 mM sodium acetate buffer (pH 5.5). All solutions were sterilized by sterile filtration.

### 2.2 SiRNAs

The following siRNA was used for gene knockdown: intercellular adhesion molecule (ICAM)–1 human with sense strand 5’-GCC UCA GCA CGU ACC UCU ATT-3’, antisense 5’-UAG AGG UAC GUG CUG AAG CTT-3’. E-selectin siRNA AF 488 was applied to test the transfection efficiency: with sense strand 5’-UUG AGU GGU GCA UUC AAC CTT-3’, antisense 5’-GGU UGA AUG CAC CAC UCA ATT-3’ (both from Eurofins MWG Operon, Ebersberg, Germany), and control nonsense siRNA (siSCR) (Qiagen, Hilden, Germany). Qiagen does not provide the sequence of their nonsilencing siRNAs but ensure that they have no homology to any known mammalian gene. This nonsilencing siRNA is validated by using Affymetrix GeneChip arrays and a variety of cell-based assays and shown to ensure minimal nonspecific effects on gene expression and phenotype.

### 2.3 Glass slide as a substrate for PEM build-up

PEMs were built-up on glass slides from Marienfeld GmbH (Lauda-Königshofen, Germany). The dimension of the slides was 10 x 10 x 1 mm. They were purified by ultrasonication (Bandelin RK 100H Sonorex, Bandelin electronic, Germany) with 2% Hellmanex solution from Hellma (Müllheim, Germany) before rinsing with ddH_2_O. Air-dried slides were sterilized in a heating furnace (Binder, Germany) at 200° C for 4 h prior using in PEM films build-up procedure.

### 2.4 Cultivation of EA.hy926

The human umbilical vein cell line EA.hy926 (LGC Standards GmbH, Wesel, Germany) was used for all cell experiments. Cells were cultured in Dulbecco’s Modified Eagle’s Medium (DMEM) high glucose containing 10% fetal calf serum, 1% Penicillin/Streptomycin, and 1% L-glutamine (gibco® by Life Technologies, Carlsbad, California). Cell number and viability was tested by a CASY cell counter (Schärfe System).

### 2.5 Complexation of PEI-siRNA particles

PEI-siRNA particles were formed by diluting siRNA (20 μM) and PEI 25 kDa in 0.15 M NaCl (Fresenius Kabi, Bad Homburg, Germany) each. For PEM-mediated transfection, 0.5 μg siRNA was used for one layer. After 10 min incubation the two solutions were mixed, shortly vortexed and span down. Within 20 min, the PEI-siRNA particles were allowed to form at RT. All PEI-siRNA particles used in the PEM build-up had an mN/P ratio of 18.75. The N/P ratio is determined by the amount of moles of primary amine groups (PEI) and the number of moles of phosphate groups of siRNA.

### 2.6 Build-up of PEM films

PEMs were built-up manually with increasing number of bilayers: PEI(HA/PEI)_2_(HA/PEI-siRNA)_3,5,7,10,12,24_. Therefore, purified and sterilized glass slides were coated using the layer-by-layer technique. Layers of HA/PEI are designated as precursor layers, whereas HA/PEI-siRNA layers are designated as bioactive layers throughout this manuscript. The first precursor layer of 750 kDa PEI was deposited by dipping the glass slide for 10 min in PEI solution. After extensively rinsing for 3 x 2 min in 0.15 M NaCl, two further precursor bilayers of HA and 25 kDa PEI (HA/PEI)_2_ were alternately added (dipping time: 10 min). Between each deposition, a washing step (3 x 2 min) with sodium acetate buffer and NaCl solution was held. Following this, bioactive layers were produced in two different ways. In the layer-by-layer method, the desired number of active layers (HA/PEI-siRNA) was deposited on precursor layers as follows: Glass slides were dipped in HA and PEI-siRNA particle solution for 10 min with washing steps as described above. In the modified layer-by-layer coating method, glass slides were dipped in HA for 20 sec and air-dried. Subsequently, PEI/siRNA particles (0.5 μg siRNA) were pipetted and not dipped onto the slide and air-dried, too. The coated slides were used for substrate mediated delivery in transfection experiments with EA.hy926 and for the release study.

### 2.7 Build-up of monolayer films

As described in the chapter before, prepared glass slides served as a substrate for the monolayer coatings in cell culture experiments. PEI-siRNA particles with either siICAM–1 or siSCR were complexed with PEI 25 kDa as described in chapter 2.5. The 10-fold amount of siRNA, PEI and NaCl was used for the total amount of 5 μg siRNA, which was also present in the 10 bilayers. The transfection solution was pipetted onto the glass slides and air-dried. Monolayer films were tested in cell culture experiments for the comparison of cell viability with PEMs.

### 2.8 PEM-mediated transfection

**Fig 1:**
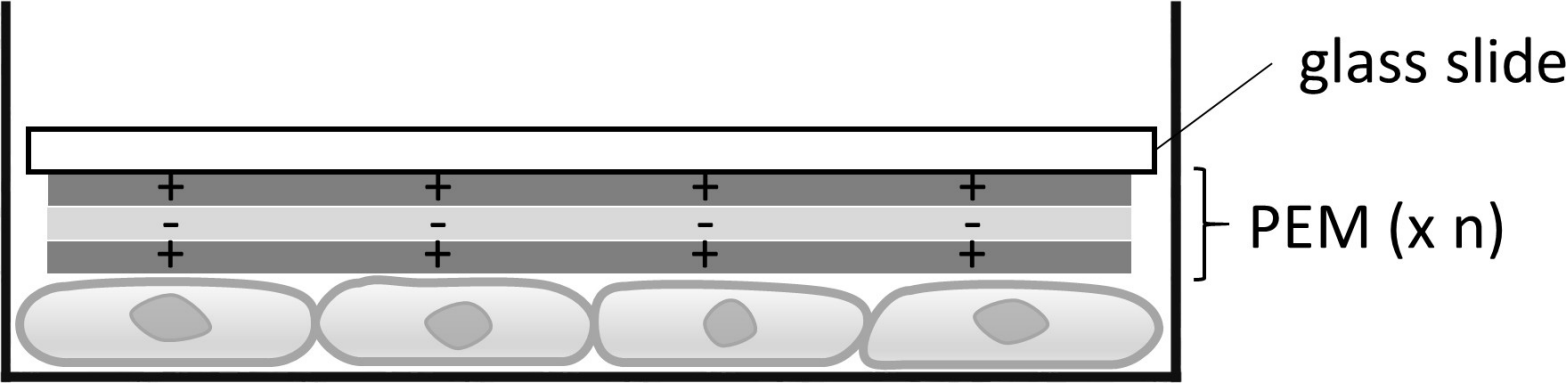
Reverse assay for cell transfection. The coated glass slide is placed on the previously seeded and cultured cells to transfect them.

Cell seeding with 1 x 10^5^ cells in a 24–well plate was prepared 48 h before transfection with PEM- or monolayer-coated glass slides. After media exchange, the coated glass slides with siRNA AF 488 and siSCR were deposited onto the cells in a so called reverse assay [52]. This assay is illustrated in Fig 1. After deposition of the glass slides 1 mL medium was added into each well and incubated for 24 h at 37°C with 5% CO_2_. Uncoated glass slides served as a negative control.

### 2.9 Flow cytometry

Transfection efficiency was determined by flow cytometry after 24 h cultivation. Glass slides were removed from the cells and they were washed and detached from cell culture plate before fixing them with 2.5% paraformaldehyde (PFA) in round-bottom special tubes for flow cytometry applications (Falcon®). Flow cytometry analysis was performed with 5000 cells/measurement (FACScan™, Becton Dickinson GmbH) and evaluated with CellQuestPro software (Becton Dickinson GmbH). Knockdown of ICAM–1 protein expression was examined as follows, cells were stimulated with 5 ng/mL tumor necrosis factor (TNF)-α (BD Biosciences, Germany) for 14 h to induce ICAM–1 expression. Immunofluorescence staining with PE mouse anti-human CD54 (BD Bioscience, Germany) was prepared before paraformaldehyde (PFA) fixing and flow cytometry analysis for 30 min at 37° C.

### 2.10 Release of PEI-siRNA AF 488 particles

Testing the PEM-mediated release of PEI-siRNA particles, complexes with 0.5 μg siRNA AF488 were formed with a mN/P ratio of 18.75. PEMs with 3, 5, 7, and 10 bilayers were built-up as described before. An uncoated glass slide and a 10 bilayer coated slide without siRNA served as controls. The slides were incubated in a 24-well plate with 500 μL PBS at 37° C. At different time points (1 h, 4 h, 24 h, and 48 h), the fluorescence intensity of the supernatant was measured with the fluorescence reader Mithras LB 940 (Berthold Technologies, Germany) with an excitation wavelength of 485 nm and an emission wavelength of 535 nm. Additionally, coated glass slides were scanned after buffer removal with 25 x 25 horizontal and vertical steps by rows, a rectangular scanning mode and a point displacement at 0.65 mm.

### 2.11 Statistics

The experiments were performed three times independently and the resulting values were tested and proven for Gaussian distribution. The values were compared by One-way ANOVA and Bonferroni correction as a Post-test. Difference between different treatment groups were determined as significant, if p < 0.05.

The analysis were performed with the statistic program GraphPad Prism 5 (GraphPad Software, La Jolla, USA).

## 3 Results

### 3.1 Reduction of ICAM–1 expression by polyelectrolyte multilayers

**Fig 2:**
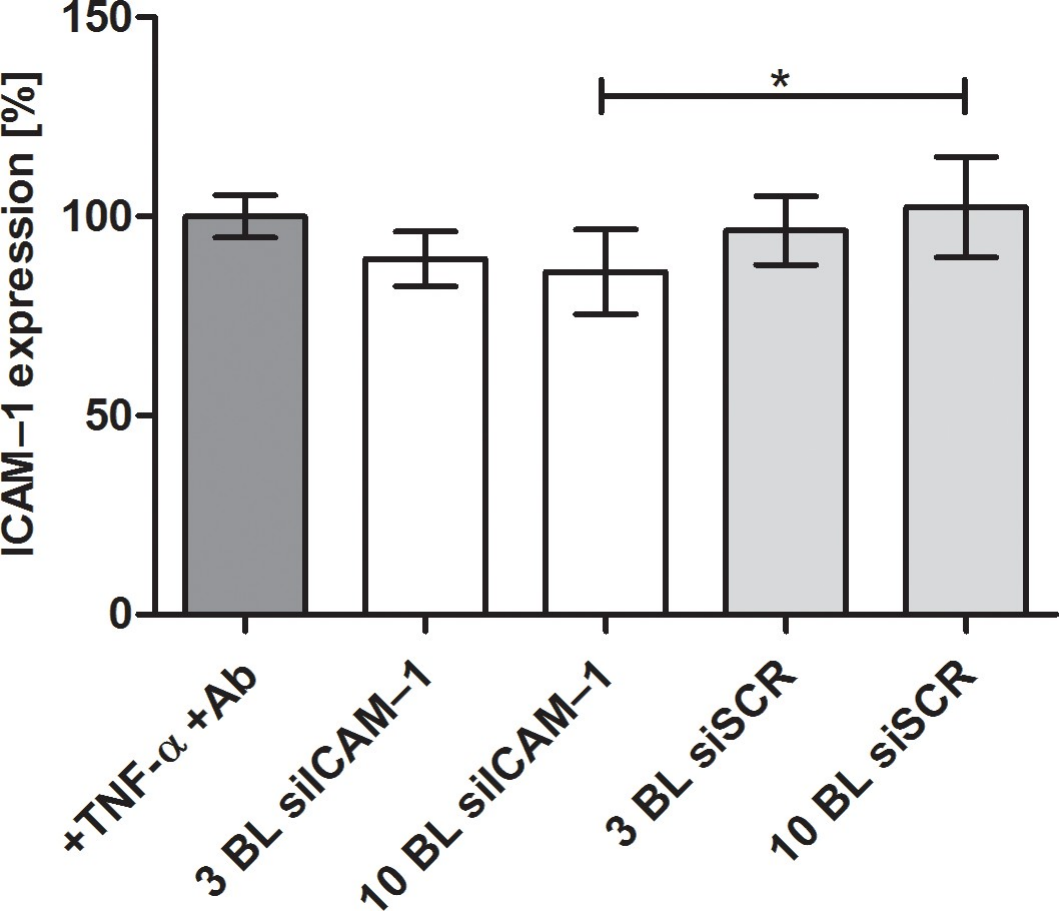
ICAM–1 expression of EA.hy926 after cultivation with PEMs. The multilayers were prepared by the dip and wash method without drying the PEI-siRNA particles as in the modified method. After 24 h of cultivation using the reverse assay, ICAM–1 expression was determined by flow cytometry and untreated but TNF-α stimulated cells were set to 100%. BL = bilayer, Ab = antibody. Each bar represents the mean ± standard error (SEM) of n = 6. * Statistical significance at p < 0.05.

PEMs were prepared by incubating in the respective solution without drying PEI-siRNA particles on the preceding layers as described in the method for modified PEMs. The PEI(HA/PEI)_2_(HA/PEI-siRNA)_3,10_ coating showed a minimal decrease of ICAM–1 expression with remaining 89% and 86% but was not significant compared to the control (Fig 2). Only the PEI(HA/PEI)_2_(HA/PEI-siRNA)_10_ and PEI(HA/PEI)_2_(HA/PEI-siSCR)_10_ coatings differed significantly from each other with 86% ICAM–1 expression and 102%, respectively.

### 3.2 Transfection efficiency of EA.hy926 mediated by PEI-siRNA AF488 modified polyelectrolyte multilayers

**Fig 3:**
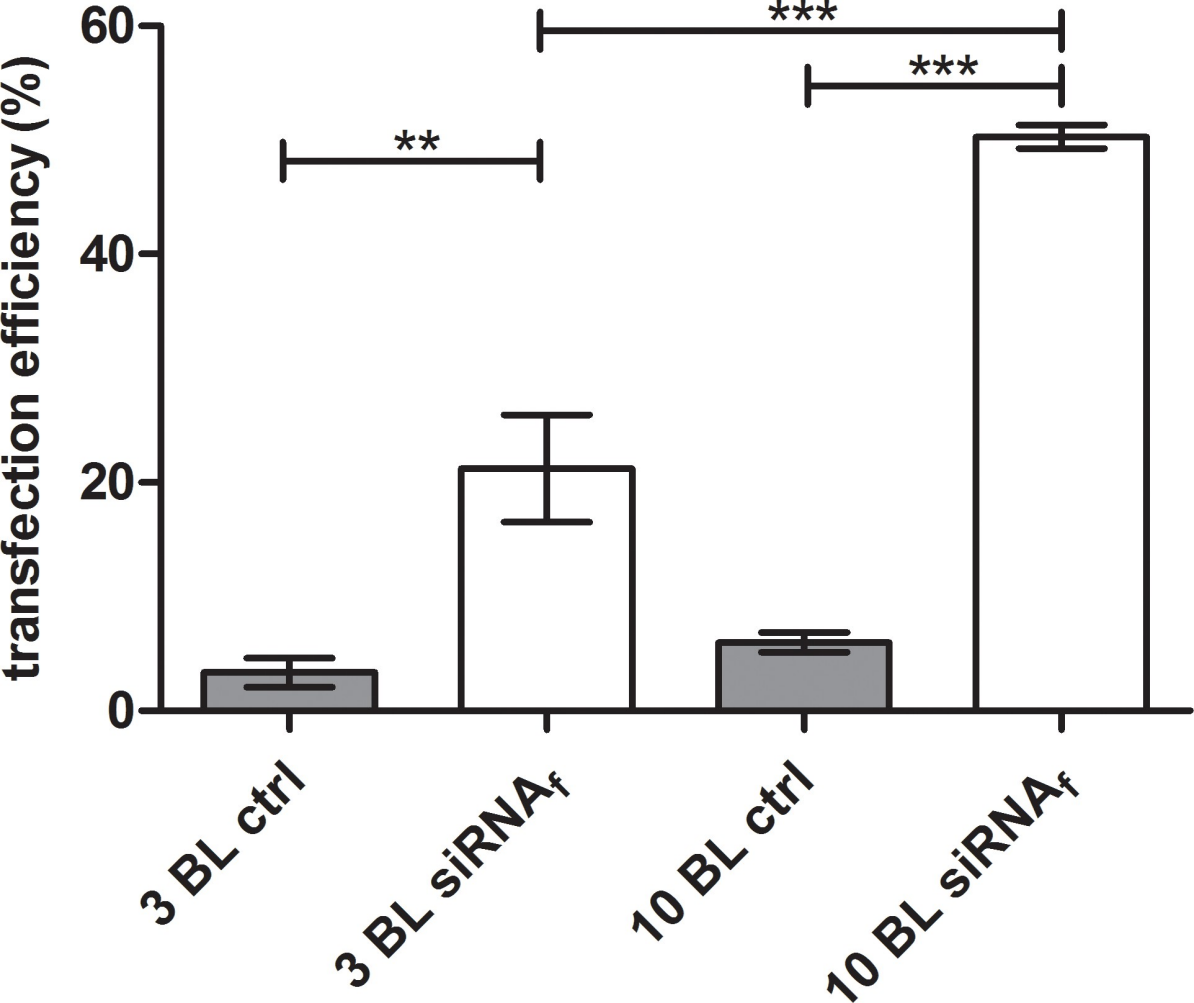
Evaluation of the siRNA uptake in EA.hy926 by flow cytometry. Cells were incubated with PEI(HA/PEI)_2_(HA/PEI-siRNA_f_)_3,10_ coated slides in the reverse assay for 24 h. The fluorescence signal of transfected cells was measured by flow cytometry. Control slides were prepared without siRNA AF 488. BL = bilayer; ctrl = control. Each bar represents the mean ± standard error (SEM) of n = 3. *** Statistical significance at p < 0.001, ** statistical significance at p < 0.01.

The efficient uptake of agents into cells is mandatory for their effectiveness and the following response of the cells. Fluorescent labeled siRNA is a suitable and smart device determining the uptake of siRNA into the cells by flow cytometry. Therefore, we examined the transfection efficiency of PEI(HA/PEI)_2_(HA/PEI-siRNA_f_)_3,10_ with incorporated siRNA AF488 and EA.hy926 cells. After 24 h incubation of coated slides on pre-cultured EA.hy926, the uptake of siRNA AF 488 particles was evaluated by flow cytometry. A transfection efficiency of 21% was measured with PEM PEI(HA/PEI)_2_(HA/PEI-siRNA_f_)_3_ and 50% with PEM PEI(HA/PEI)_2_(HA/PEI-siRNA_f_)_10_ (Fig 3). Both active PEMs with siRNA AF 488 showed significant higher transfection efficiency values than the control slides PEI(HA/PEI)_2_(HA/PEI)_3,10_. Furthermore, the PEM PEI(HA/PEI)_2_(HA/PEI-siRNA_f_)_10_ with 10 bilayers achieved a significantly higher transfection efficiency than PEI(HA/PEI)_2_(HA/PEI_f_)_3_.

### 3.3 ICAM–1 expression after transfection with modified polyelectrolyte multilayers

**Fig 4:**
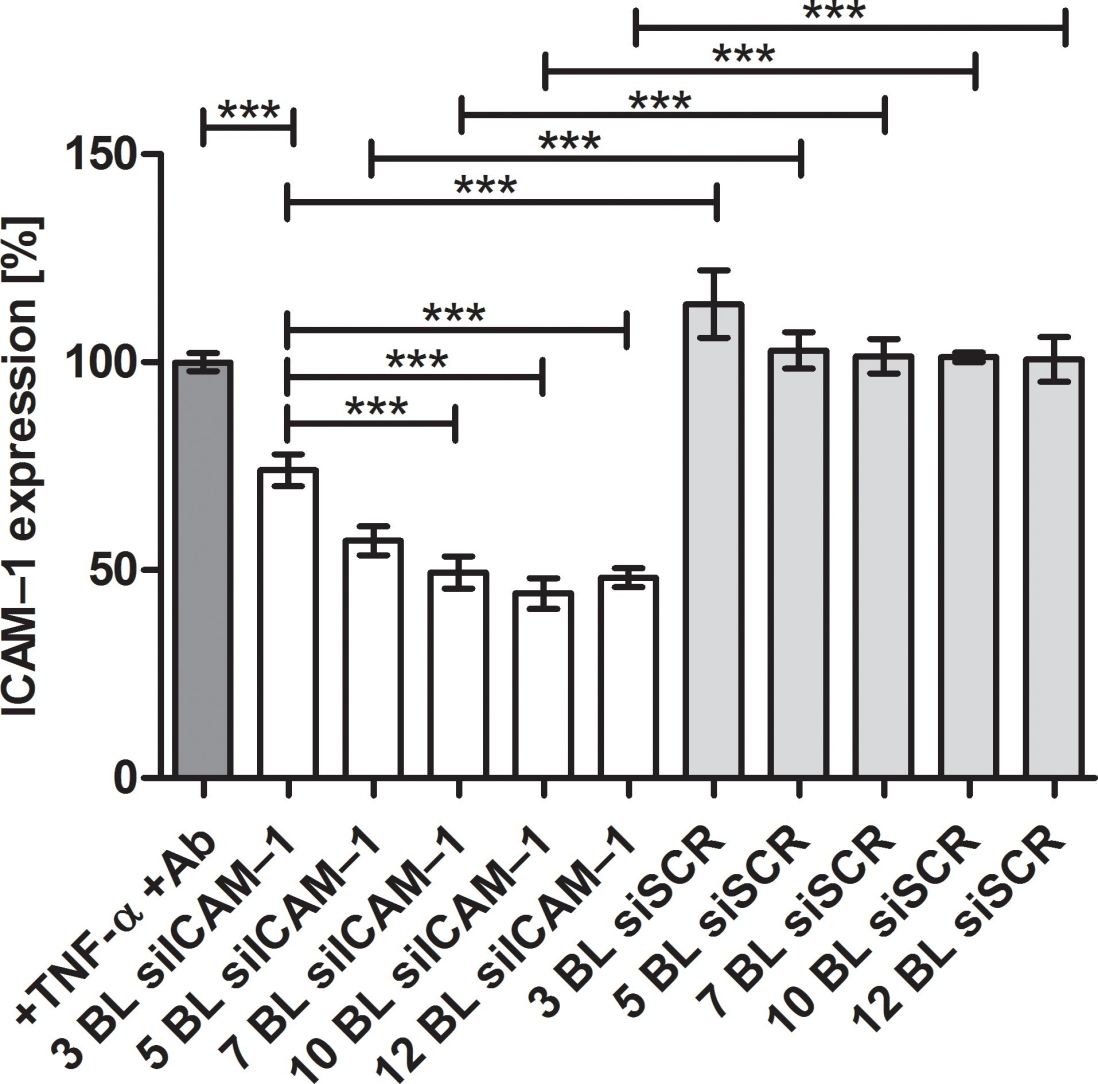
ICAM–1 expression after 24 h reverse assay with EA.hy926. Cells were cultivated with PEI(HA/PEI)_2_(HA/PEI-siRNA)_3,5,7,10,12_ coated glass slides for 24 h. Thereafter cells were stimulated with TNF-α for 14 h. After ICAM–1 antibody staining, fluorescence signal was determined by flow cytometry. Untreated but TNF-α stimulated cells were set to 100% and the results of the treatment groups represent the expression of ICAM–1 receptor in %. Ab = antibody, BL = bilayer. Each bar represents the mean ± standard error (SEM) of n = 5. *** Statistical significance at p < 0.001.

To determine the most effective amount of bilayers for gene knockdown, we tested 3, 5, 7, 10, 12 bilayers of (HA/PEI-siRNA)_n_. The results show that the desired ICAM–1 expression decrease was achieved in the samples with increasing number of layers and amount of siICAM–1. PEI(HA/PEI)_2_(HA/PEI-siICAM–1)_3,5,7,10,12_ showed significant reduction of ICAM–1 expression in comparison to control cells which were stimulated with TNF-α and antibody treated. The highest knockdown was achieved with 10 bilayers and remaining 44% ICAM–1 expression (Fig 4). 7 and 12 bilayer revealed similar ICAM–1 expression levels with 49% and 48% respectively. The ICAM–1 expression of control PEMs with siSCR were at the expression level of the control cells.

### 3.4 The effectiveness of modified polyelectrolyte multilayers for ICAM–1 knockdown

Two sizes of PEM bilayers PEI(HA/PEI)_2_(HA/PEI-siICAM–1)_10,24_ were evaluated for the delivery of siICAM–1 and the following knockdown of the ICAM–1 receptor in EA.hy926. 10 bilayers were chosen due to the results in 2.3 where 10 bilayers provoked the highest ICAM–1 knockdown. Additionally, a high amount of PEMs with 24 bilayers was tested to verify if a significant increase in bilayers and thus total siRNA levels could result in higher knockdown or prolonged release. First, cells were incubated with PEI(HA/PEI)_2_(HA/PEI-siICAM–1)_10,24_ and PEI(HA/PEI)_2_(HA/PEI-siSCR)_10,24_ for 24 h. Afterwards the same PEM coated glass slides were incubated with new pre-cultured cells for another 24 h.

**Fig 5:**
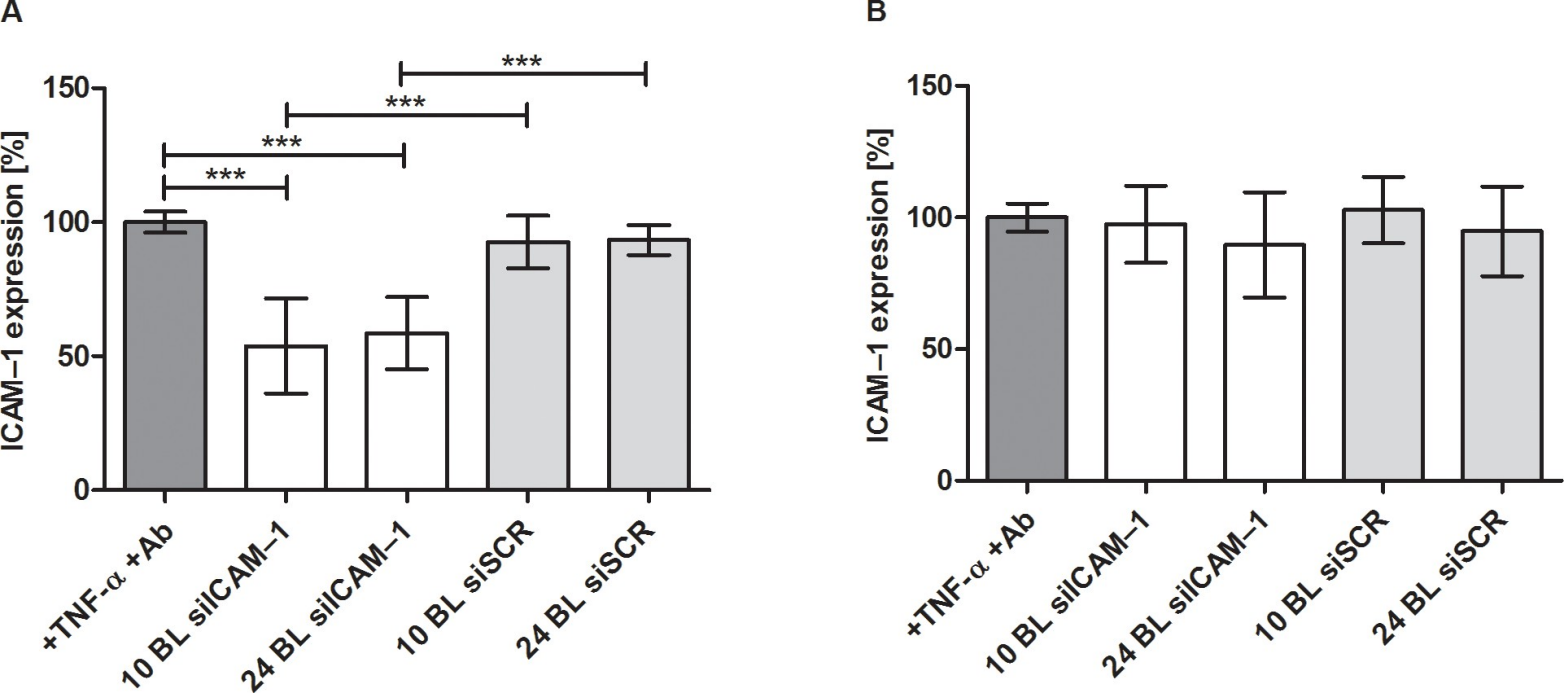
ICAM–1 expression after 24 h and following 24 h after re-cultivation of the same PEMs with new seeded EA.hy926. A: After 24 h reverse assay with 10 and 24 bilayers, cells were analyzed by flow cytometry and untreated but with TNF-α stimulated cells were set to 100%. B: The same 10 and 24 bilayer were cultivated for another 24 h with new pre-cultivated EA.hy926 and analyzed as described in A. Ab = antibody, BL = bilayer. Each bar represents the mean ± standard error (SEM) of n = 6. *** Statistical significance at p < 0.001.

The ICAM–1 expression was significant reduced by 10 and 24 bilayers after the first 24 h of incubation (Fig 5A). The 10 bilayers achieved a slightly higher ICAM–1 knockdown (5%) compared to 24 bilayers. Re-cultivation of the same PEMs for 24 h did not cause any significant knockdown of the ICAM–1 receptor in fresh cultured cells (Fig 5B).

### 3.5 Release of siRNA AF 488 from modified polyelectrolyte multilayers

**Fig 6:**
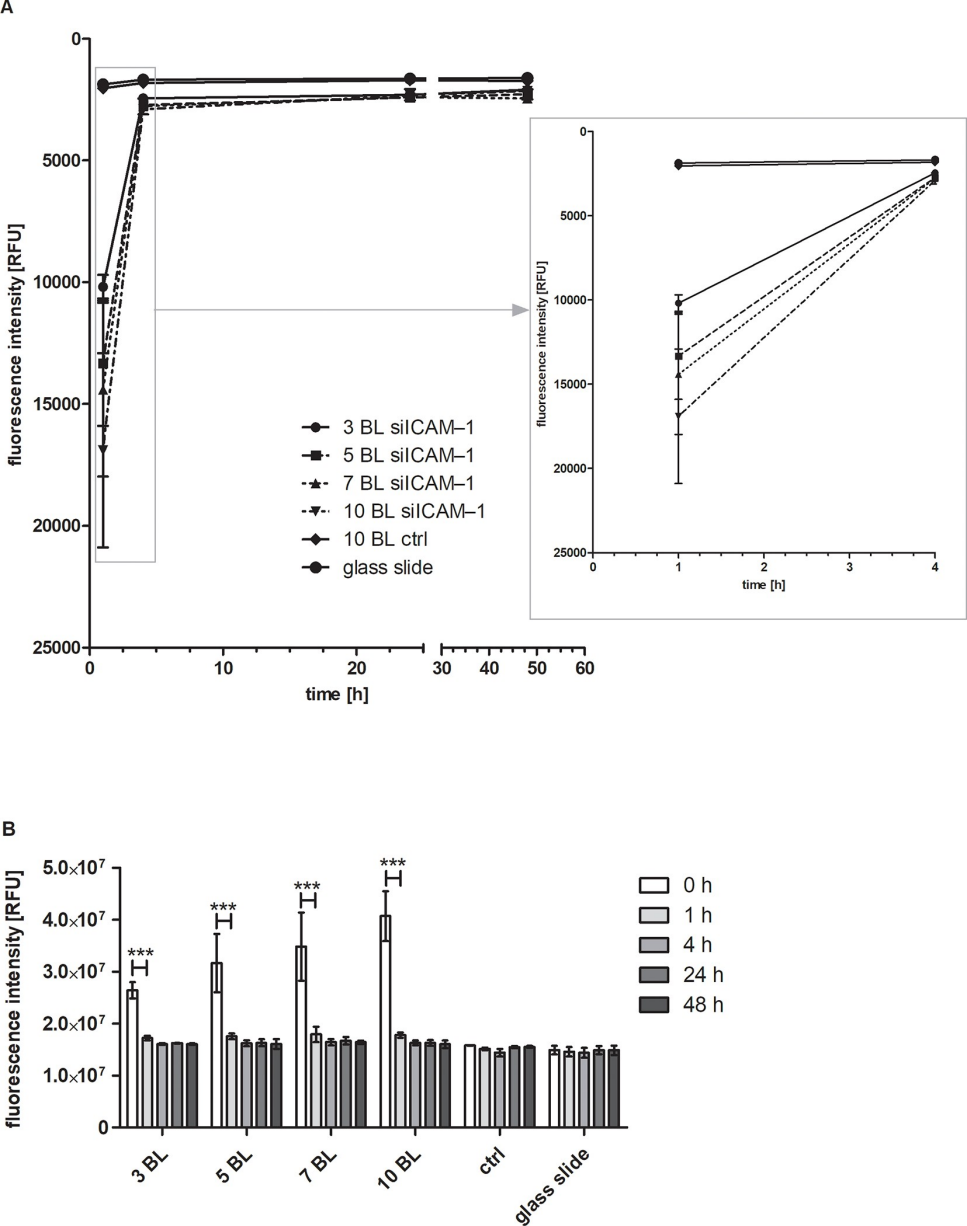
Release of siRNA AF 488 from PEMs within 48 h. A: Release of siRNA AF 488 by measurement of the supernatant. The fluorescence intensity of the supernatants was measured after 1, 4, 24, and 48 h incubation in PBS with a fluorescence reader at 485 nm excitation and 535 nm emission wavelength. In the right box an enlarged section of the period 1 to 4 h is shown. Each bar represents the mean ± standard error (SEM) of n = 3. B: Glass slide scan for release control. After the removal of the supernatant, glass slides were scanned with a resolution of 25 x 25 measurement points, at the same wavelengths as described before. The sum of all measurement points = fluorescence intensity, was determined at different timepoints. BL = bilayer; ctrl = control. Each bar represents the mean ± standard error (SEM) of n = 3. *** Statistical significance at p < 0.001.

The release of siRNA AF 488 (siRNA_f_) from PEMs PEI(HA/PEI)_2_(HA/PEI-siRNAf)_3,5,7,10_ was determined after 1, 4, 24, and 48 hours incubation in PBS (Fig 6A). The supernatant was measured with a fluorescent reader. After 1 h of incubation, the highest relative fluorescence unit (RFU) signal (16,899) was measured with 10 bilayers. The values of the RFU were getting less with the decrease of bilayers: 14,409 RFU with 7 bilayers, 13,325 RFU with 5 bilayers and 10,184 with 3 bilayers. Both control glass slides, the uncoated and the PEI(HA/PEI)_2_(HA/PEI-siSCR)_10_, showed low fluorescence signals with 1,874 and 2,034 RFU, respectively. After 4 hours incubation, the fluorescence signals of PEI(HA/PEI)_2_(HA/PEI-siRNA_f_)_3,5,7,10_ decreased into the range of both controls with 2,900, 2,779, 2,731, and 2,444 RFU for 10, 7, 5, and 3 double layers, respectively. The following RFU values of PEI(HA/PEI)_2_(HA/PEI-siRNAf)_3,5,7,10_ glass slides were on the RFU level of the control and uncoated glass slides after 24 and 48 h.

In order to determine the total amount of siRNA AF 488 applied during PEM coating, the glass slides were scanned immediately after the coating procedure (time point 0 h) and the fluorescence was analyzed. Furthermore, the glass slides were scanned after the incubation times (1 h, 4 h, 24 h, and 48 h) after removal of the supernatants (Fig 6B). Highest fluorescence intensity sum was found at timepoint zero directly after PEM coating with 2.6 x 10^7^ RFU for 3 bilayers, 3.2 x 10^7^ RFU for 5 bilayers, 3.5 x 10^7^ RFU for 7 bilayers, and 4.1 x 10^7^ RFU 10 bilayers. After 1 h incubation all intensity values of 3, 5, 7, and 10 bilayers were significantly reduced to 1.7 x 10^7^ and 1.8 x 10^7^ RFU and were nearly at control level, which was 1.5 x10^7^ RFU. From the time point 4 h there was no significant change in the fluorescence intensity of siRNA AF 488-containing PEM and all samples had similar RFU values.

### 3.6 Influence of different siRNA coatings on cell viability

**Fig 7:**
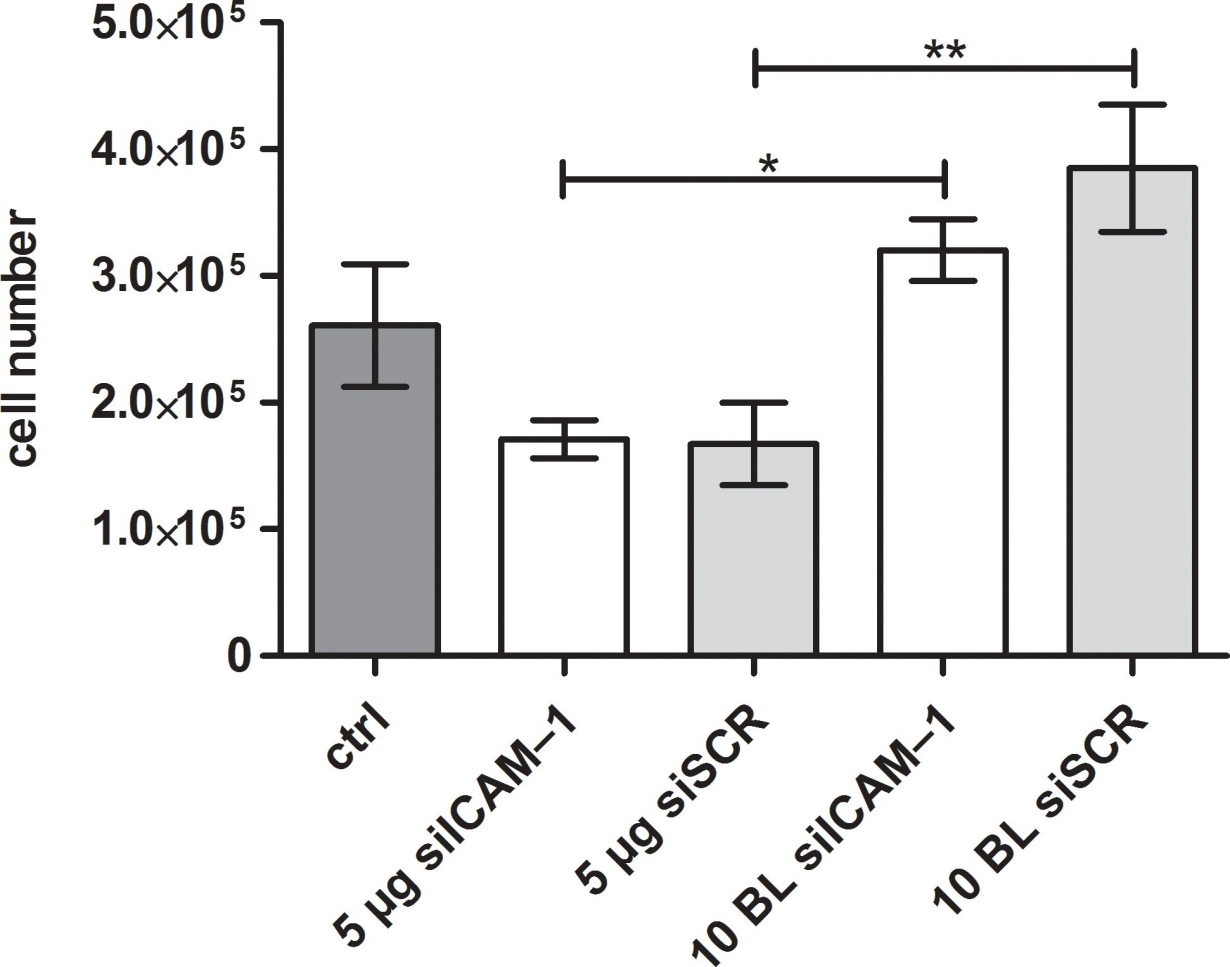
Cell number of EA.hy926 after incubation with PEI-siRNA particles and PEMs. EA.hy926 were incubated with different coated glass slides for 24 h and then tested for cell number after detachment with a CASY cell counter. Cells cultivated with an uncoated glass slide served as a control. 10 BL PEMs were compared with the monolayer of PEI-siRNA with the same siRNA amount (5 μg). BL = bilayer, ctrl = control. Each bar represents the mean ± standard error (SEM) of n = 3. *** Statistical significance at p < 0.001, ** statistical significance at p < 0.01, * statistical significance at p < 0.005.

Polyelectrolyte multilayers, siRNA and transfection agents may influence the cell viability during the cultivation. The influence of these parameters was tested by determination of cell number and viability of EA.hy926 with a CASY cell counter. It is of interest whether the PEMs are superior to the monolayer system in terms of cell viability. To determine the compatibility of PEM-coated slides in comparison to the same amount of PEI-siRNA in a monolayer system (hereby, the same amount of siRNA and PEI which was used for 10 bilayers was used to dry into a monolayer), cells were analyzed after 24 h reverse assay. PEM of PEI(HA/PEI)_2_(HA/PEI-siICAM–1)_10_ showed a significant higher cell number of 3.2 x 10^5^ in comparison to the monolayer with the same siICAM–1 amount and the cell number 1.7 x 10^5^ (Fig 7). The same result can be seen in the samples with siSCR. Again, the cell numbers are significantly different from each other and the PEM of PEI(HA/PEI)_2_(HA/PEI-siSCR)_10_ had a significantly higher number on 3.9 x 10^5^ cells than the monolayer with 1.7 x 10^5^ cells. The control sample where cells were cultivated with uncoated glass slides showed a cell number of 2.6 x 10^5^ and is even lower than the cell number of PEMS.

## 4 Discussion

Substrate-mediated gene silencing by RNAi has great potential for therapeutic approaches where local effect is desired. A sophisticated and today well developed method is the LbL technique, where a multilayer system can be built with different biomaterials of natural or synthetic origin with opposite charges. The structure of these PEMs allows the embedding of siRNA nanoparticles for gene silencing. To transport the siRNA, a cationic polymer like PEI can be used so that the siRNA, protected from enzymatic degradation, enters the cell for transfection.

The purpose of this study was the development of an siRNA-eluting coating using the LbL technique for gene silencing of the adhesion molecule ICAM–1 that plays a key role in the adhesion of leukocytes during leukocyte diapedesis at inflammatory sites [53]. Two different build-up methods with either dipping or drying (modified PEMs) steps were tested for HA/PEI PEMs. The PEMs were characterized for siRNA release, for cell viability, for transfection efficiency and ICAM–1 knockdown. Therefore, the reverse assay [51, 54] was applied for all cell culture assays to evade the possible influence of mechanical properties of the biomaterials HA and PEI on cell growth.

The build-up of PEMs PEI(HA/PEI)_2_(HA/PEI-siRNA)_n_ was initially prepared by the dip and wash method. The ICAM–1 expression results showed that neither 3 bilayers (1.5 μg siRNA) nor 10 dipped bilayers (5 μg siRNA) could induce uptake of siRNA after 24 h (Fig 2). The small decrease in ICAM–1 expression with 3 and 10 bilayers suggests that only small amounts of siRNA particles were incorporated into PEMs which results in a low transfection efficiency. We suppose that during the dipping step with PEI-siRNA particles, the incubation time was insufficient to bind to the hyaluronic acid. A recent study of Holmes and Tabrizian showed an efficient LbL system with glycol-chitosan/HA PEMs where DNA lipoplexes were adsorbed for 2 h followed washing and further adsorption steps. Herein, NIH3T3 and HEK293 cells were successfully transfected with different amount of DNA (2; 4; 6 μg) [22].

Consequently, we modified the process of the LbL method by omitting the washing steps after the precursor layers and dried the functional bilayers of PEI-siRNA and HA on the platelets. The purpose of the modification was to change the build-up of the PEMs as little as possible. With the modified drying method of PEM assembly, a coating could be established that released PEI-siRNA particles which were taken up by the cells through substrate-mediated transfection. The transfection efficiency showed the significant uptake of siRNA with 3 and 10 bilayers compared to control, moreover 10 bilayers provoked with 50% a significantly higher transfection efficiency compared with 3 bilayers with 21% (Fig 3). The results of ICAM–1 expression underlined the effectiveness of the modified method, where ICAM–1 expression could be significantly reduced up to 44% with 10 bilayers in comparison to the TNF-α/Ab control and the associated siSCR control (Fig 4). The 3, 5, 7, and 12 bilayers also showed significant ICAM–1 reduction in comparison to both controls. Within the experimental siICAM–1 series, there was also a significant difference between the 3 bilayers and the 7, 10 and 12 bilayers. This result indicates that an augmentation of bilayers up to 10 is meaningful for reaching the highest reduction of ICAM–1 expression. This was also proved with the knockdown experiment with 10 and 24 bilayers in comparison (Fig 5A). An increase in the number of bilayers, and the associated additional increase of siICAM–1 in the layers cause no further reduction in ICAM–1 expression. We might suppose with regard to this result either transfected cells were saturated with siRNA particles from the bilayers and consequently were no longer capable for siRNA uptake or the build-up of PEMs has reached its maximum after 10 bilayers in our study. In a previous preliminary study it was observed that also the application of 15 bilayers had no higher reduction of the ICAM–1 expression, and the value was between that of the 10 and 12 bilayers.

The decrease of the ICAM–1 expression (Fig 4) in response to the increase in the number of layers indicates that the layer build-up has taken place successfully using the drying method. The glass slide scanning dates at time point zero corroborate these results showing an increase in fluorescence intensity with an increase in bilayers (Fig 6B) as well as the release results after 1 h incubation (Fig 6A). Consequently, these results suggest that the amount of siRNA_f_-PEI has been incorporated in every bilayer.

The duration of siRNA release for substrate-mediated transfection is of particular importance for the transfer of the system to medical devices, such as stents or balloon catheters in atherosclerosis therapy. For long-term release of drugs, the stent is a suitable medical device in percutaneous transluminal coronary angioplasty (PTCA) and is called drug eluting stent (DES) [55, 56]. However, fast-release systems are of particular interest in medical interventions where no medical device remains in the body that releases a drug in the long-term. Currently, coated balloon catheters are used for the treatment of atherosclerosis with for instance paclitaxel, which is intended to prevent or ameliorate restenosis [57–59]. This fast-release coatings may be beneficial to deliver the drug during treatment to the site of action. The release experiment over 48 h (Fig 6A) showed on the basis of the fluorescence intensity that almost the entire amount of PEI-siRNA_f_ particles was released from the PEM within the first hour. After 4 hours the values of all BL approximated to the values of the control glass slide. The glass slide scanning results underlined this finding. At the measuring point 1 h a significant decrease in the fluorescence intensity of all siRNA_f_ bilayers was observed. The fluorescence intensity of these samples continued to decline at point 4 h, 24 h, and 48 h with an approach to the control values (Fig 6B). In conclusion, an initial burst release followed by minor siRNA_f_ release could be achieved with the modified drying build-up method. The re-cultivation of the glass slides PEI(HA/PEI)_2_(HA/PEI-siICAM–1/siSCR)_10_,_24_ for another 24 h confirmed the burst release because no significant decrease of ICAM–1 expression could be achieved after their deposition on pre-cultured EA.hy926 (Fig 5B).

Next to the transfection efficiency, the cell viability is of great importance for substrate-mediated transfection with PEM systems. Therefore, we used the reverse assay with 24 h cultivation of EA.hy926 and counted the cell number with the CASY^®^ system. For cells that were cultivated with a monolayer containing 5 μg PEI-siICAM–1 or 5 μg PEI-siSCR, the cell numbers were reduced in comparison to the control cells incubated with an uncoated glass slide. On the other hand the cells that were cultivated with the PEM PEI(HA/PEI)_2_(HA/PEI-siICAM–1/siSCR)_10_, the cell numbers were higher than the control (Fig 7). The direct comparison between monolayer and the PEM system revealed significant higher cell numbers of EA.hy926 cultivated with PEI(HA/PEI)_2_(HA/PEI-siICAM–1/siSCR)_10_. We presume that these findings are due to the HA which is incorporated in the PEM system but is not present in the monolayer. Han et al. discovered in their study the positive effect of HA regarding the cytotoxicity of PEI in their HA-PEI delivery system. They detected that the combination of HA-PEI provoked less cytotoxicity than PEI alone [48]. Furthermore, McKee et al. described an anti-inflammatory effect of high molecular weight HA which was used in our and Han’s study [45]. We suppose that this effect is able to protect the cells from PEI cytotoxic side effects.

## Conclusion

In this study we established a multilayer system consisting of the polyelectrolytes PEI and HA by an LbL technique which successfully transfected cells with siRNA by substrate-mediated transfection. We proofed that the classic LbL dipping method is not capable to provoke a significant reduction of ICAM–1 expression after 24 h. Therefore, we modified the LbL method by a new developed drying method and achieved significant ICAM–1 expression reduction from 3 BL, 5 BL, 7 BL, 10 BL, up to 12 BL. The coating with 10 BL showed the highest knockdown with 44% remaining ICAM–1 expression and transfection efficiency with fluorescent labeled siRNA of 50%. An increase in the number of bilayer up to 24 did not yield any further reduction of ICAM–1 expression. The release study of the PEMs demonstrated that there is a burst release within the first hour followed by a minor release. The re-cultivation of previously laid out 10 and 24 BL PEMs confirmed this result. We demonstrated by counting the viable cells that the PEM system influences the cell number positively in comparison to the monolayer system, where cell number was reduced significantly. This new developed modified LbL coating could be easily adapted for medical devices where a fast substrate-mediated transfection of cells is desired, e.g. balloon catheter during PTCA. Therefore, we see great potential for an application in the prevention of restenosis on molecular level after a balloon catheter intervention via PTCA The high knockdown results make the construction interesting for further developments in the field of local gene delivery systems.
